# NL reading skills mediate the relationship between NL phonological processing skills and a foreign language (FL) reading skills in students with and without dyslexia: a case of a NL (Polish) and FL (English) with different degrees of orthographic consistency

**DOI:** 10.1007/s11881-019-00181-x

**Published:** 2019-07-08

**Authors:** Marta Łockiewicz, Martyna Jaskulska

**Affiliations:** Institute of Psychology, Social Sciences Faculty, University of Gdansk, Bażyńskiego 4, 80-952 Gdańsk, Poland

**Keywords:** Dyslexia, English as a Foreign Language, Orthographic consistency, Phonological processing, Reading

## Abstract

The aim of our study was to examine the relationship between NL (Native Language: Polish) phonological processing skills (verbal and phonological short-term memory, phoneme segmentation and blending, rapid automatised naming (RAN)) and the accuracy and fluency of NL and English as a Foreign Language (EFL) word and nonword decoding and word recognition skills of Polish students with and without dyslexia. Sixty-three (45%) high school and junior high school students with and 78 (55%) without dyslexia participated. We found that dyslexia, years of studying EFL at school and privately, NL phoneme blending and RAN predicted word reading accuracy in EFL, and dyslexia, years of studying EFL privately, and NL RAN predicted EFL word reading fluency. Dyslexia and NL phoneme blending predicted the accuracy, and NL RAN—the fluency of EFL nonword decoding. These findings confirm that difficulties in FL acquisition result from NL phonological processing deficits, characteristic of dyslexia. Our results also showed relationships between NL phonological processing and EFL reading that were analogical to the ones observed for NL. The pattern of relations between NL phonological processing, NL reading, and EFL reading was similar for reading fluency, but not for reading accuracy in the compared groups. Both NL phonological processing and NL reading facilitated EFL reading, though it was more conspicuous in the control group, which suggests that readers with dyslexia benefit less from their NL reading skills when learning to read in FL.

## Introduction

Linguistic interdependence theory states that the development of language and literacy competence in a foreign language (referred hereafter in the paper as FL) stems from the competence level already developed in NL (a mother tongue) at the time when FL exposure begins (Cummins, 1979). The Linguistic Coding Differences Hypothesis (LCDH) indicates specifically a cross-linguistic transfer of phonological aptitudes and difficulties (Sparks, Patton, Ganschow, & Humbach, 2009; Sparks, Patton, Ganschow, Humbach, & Javorsky, 2006). Namely, effective FL learning results from better phonological and orthographic, but not semantic, NL skills, which was observed for college (Ganschow, Sparks, Javorsky, Pohlman, & Bishopmarbury, 1991) and high school students, including students with learning disabilities (Sparks, Ganschow, Javorsky, Pohlman, & Patton, 1992). Moreover, NL literacy skills predict FL proficiency. For students learning English as NL, FL (Spanish, French, German) proficiency in year 10 was best predicted by reading readiness (rhyming, letter-sound relationships, NL word decoding) in year 1 students, and by NL reading (word and nonword decoding, reading vocabulary, reading comprehension) in year 5 students (Sparks et al., 2006). Similarly, college students’ NL nonword decoding ability was related to FL literacy skills (FL vocabulary, grammar, and reading comprehension) (Meschyan & Hernandez, 2002).

For learning EFL, the meta-analysis by Melby-Lervåg and Lervåg (2011) demonstrated NL-EFL cross-linguistic transfer for decoding and phonological awareness skills in the immersion context (schooling in EFL for at least 4 h per day). Phonological awareness in 5–6-year-old Norwegian (Helland & Morken, 2016) native speakers predicted EFL word reading at the age of 8 and 10, and EFL word reading, spelling, and translation at the age of 11, respectively. NL and EFL phonological short-term memory (as measured with nonword repetition) predicted EFL vocabulary learning in Finnish elementary school students (Service, 1992). In Italian children, year 6 calculation skills and NL reading comprehension were the best predictors of EFL dictation and exercises performance in year 8, respectively (Ferrari & Palladino, 2012). In Dutch secondary school students, RAN, but not phoneme awareness, uniquely influenced EFL speeded word and text reading (Morfidi, Leij, Jong, Scheltinga, & Bekebrede, 2007). Moreover, speeded word reading in NL predicted an analogical task and text reading in EFL. These findings suggest that NL phonological processing skills influence both NL and EFL reading, and NL reading influences EFL reading. However, we found no study that would examine the mediating role of NL literacy between NL phonological processing skills and EFL literacy.

Dyslexia is a specific learning disability that should affect FL literacy acquisition due to characteristic impairments in NL phonological processing deficits: phonological awareness, short-term memory (Hoien, Lundberg, Stanovich, & Bjaalid, 1995; Snowling, 2000), and RAN (Wolf, Bowers, & Biddle, 2000). In fact, problems of students with dyslexia in FL reading have been demonstrated for several languages (Lindgren & Laine, 2011; Palladino, Bellagamba, Ferrari, & Cornoldi, 2013; van Sette et al., 2017), though NL learning difficulties do not equal FL learning problems (Sparks, 2006).

The difficulty of word and nonword reading depends also on the orthographic consistency of a given language (Seymour et al., 2003). English and Polish phonology differ in their phoneme repertoires. For reading, English orthography is more inconsistent as compared with Polish (Awramiuk, 2006). Thus, Polish readers of EFL struggle with phoneme-grapheme correspondence irregularities which are practically absent in their NL.

This study is a part of a larger project that aims to investigate FL language learning of learners of a semi-consistent NL to an inconsistent (English) orthography in Polish adolescents with and without dyslexia. In related studies, we found that NL verbal short-term memory and access to mental lexicon predicted EFL vocabulary in Polish junior high school students. This relationship was more conspicuous in the control group without dyslexia (Łockiewicz & Jaskulska, 2015). Moreover, Polish high school students with dyslexia read EFL actual words and nonwords less accurately and more slowly, and had a more limited EFL vocabulary, as compared with their peers without dyslexia (Łockiewicz & Jaskulska, 2016). NL and FL reading speed and accuracy correlated positively, which was more noticeable in the control group. Both high and junior high school students with dyslexia, as compared to the participants without dyslexia, made more spelling, but not grammar, errors when writing a short essay in EFL (Łockiewicz & Jaskulska, 2019). Moreover, for students with dyslexia, years of studying EFL at school and privately, and NL phonological processing predicted their EFL spelling, while NL spelling mediated the relationship between NL phonological processing and EFL spelling, but only in the control group (Łockiewicz & Jaskulska, 2018).

The present study combines evidence from the two databases (for high and junior high school students) used in the aforementioned papers. The unique focus of the current research was on the relationship between NL phonological processing skills (short-term verbal and phonological memory, phoneme segmentation and blending, RAN) and EFL word and nonword decoding and word recognition skills of Polish students with and without dyslexia, which had not been examined empirically before. We concentrated on the link between NL phonological processing skills and the fluency and accuracy of decoding and word recognition skills in EFL, as these are the core symptoms in dyslexia (Lyon, Shaywitz, & Shaywitz, 2003), and, according to the LCDH, deficits in the phonological code cause difficulties in FL learning (Sparks et al., 2009; Sparks et al., 2006). Word decoding and recognition depend on phonological processing skills: phonological awareness, rapid automatised naming (RAN), and verbal short-term memory (Krasowicz-Kupis, 2008; Wagner & Torgesen, 1987). Thus, we decided to examine this set of aforementioned variables, which are regarded as core in the development of and proficiency in NL and FL reading to investigate if and how they also contribute to FL reading of Polish students learning EFL. In the mediation analysis, we examined the reading variables separately, as word and nonword decoding and word recognition skills depend on partially different underlying mechanisms (Coltheart, 2006). Thus, through these separate analyses, we could have assessed more precisely the relationship between NL phonological processing and both word and nonword decoding and word recognition strategies in adolescent readers with and without dyslexia. We were interested in examining if NL word and nonword decoding and word recognition skills mediate the relationship between NL phonological processing and EFL word and nonword decoding and word recognition strategies in adolescent readers with and without dyslexia, as abovementioned studies identified the predictive function of NL literacy skills on FL literacy skills (Melby-Lervåg & Lervåg, 2011; Meschyan & Hernandez, 2002; Sparks et al., 2006), and our participants had substantially more practice and instruction in NL than EFL literacy. Specifically, reading instruction at school started approximately 2 years earlier than EFL classes and was more extensive. Thus, the novel contribution of our study is the comparison of the relationship between NL phonological processing, NL, and EFL word and nonword decoding and word recognition skills in two diverse groups: both readers with dyslexia and without dyslexia.

## Method

### Participants

Sixty-three (45%) secondary and junior secondary school students with dyslexia and 78 (55%) without dyslexia participated, all native speakers of Polish. The groups were matched for gender and educational level, age, and IQ (Table 1). As the participants had studied English for 8 years on average, we assumed that their EFL fluency allowed for adequate demonstration of differences between the compared groups. However, more students with (36 persons (57%)) than without dyslexia (32 persons (41%)) took private tutoring instruction outside school (*χ*^2^(1) = 3.626, *p* = .057), which lasted for a longer time. Such instruction usually lasts an hour weekly. The results of 2 students were excluded due to their longer stay abroad (over 6 months).

**Table 1:** Descriptive characteristic of the compared groups

	Dyslexic				Nondyslexic				
	Female		Male		Female		Male		
	H	JH	H	JH	H	JH	H	JH	
Gender and educational level^a^	19 (30)	8 (13)	25 (40)	11 (17)	21 (27)	21 (27)	28 (36)	8 (10)	*χ*^2^(1) = .01, *p* = .937
Age^b^	16.44 (18.19)				16.2 (19.28)				*t*(138) = 0.88, *p* = .386
IQ^c^	51.48 (4.06)				51.45 (4.64)				*t*(139) = 0.04, *p* = .971
English instruction at a state school^b^	8.52 (1.64)				8.32 (2.31)				*t*(131.37) = 0.59, *p = .*555
English instruction privately^b^	3.29 (3.58)				1.55 (2.64)				*t*(100.987) = 3.11, *p = .*002
Reading in Native Language (Polish): accuracy and fluency^c^									
Nonwords read incorrectly	10.40 (4.81)				6.94 (3.99)				*t*(139) = 4.67, *p* ≤ .001, *d* = 0.79
Number of nonwords read within 1 min.	48.63 (10.25)				57.92 (11.37)				*t*(137.281) = 5.09, *p* ≤ .001, *d* = 0. 87
Reading in EFL: accuracy and fluency^c^									
Words read correctly	51.94 (13.35)				58.04 (10.93)				*t*(116.979) = 2.91, *p* = .004, *d* = 0. 54
Number of words read within 1 min.	64.65 (9.72)				68.04 (4.60)				*t*(82.566) = 2.53, *p* = .013, *d* = 0. 56
Nonwords read correctly	17.85 (3.56)				21.46 (4.63)				*t*(136.972) = 5.19, *p* ≤ .001, *d* = 0. 89
Time of nonword reading (in sec.)	37.92 (11.96)				33.50 (8.72)				*t*(105.923) = 2.43, *p* = .017, *d* = 0. 47
L1 phonological processing^c^									
Verbal short-term memory	12.71 (2.09)				13.45 (2.97)				*t*(136.594) = 1.72, *p* = .088, *d* = 0.29
Phonological short-term memory	8.90 (2.76)				9.32 (2.94)				*t*(139) = 0.86, *p* = .393, *d* = 0.15
Phoneme segmentation	6.10 (1.34)				6.12 (1.28)				*t*(139) = 0.91, *p* = .928, *d* = 0.15
Phoneme blending	3.38 (1.56)				4.28 (1.82)				*t*(139) = 3.12, *p* = .002, *d* = 0.53
RAN	88.52 (20.32)				69.04 (17.68)				*t*(139) = 6.09, *p* ≤ .001, *d* = 1.03

*H* high school, *JH* junior high school

^a^Actual figures given (% in parenthesis)

^b^Mean figures in years given (*SD* in months in parenthesis)

^c^Mean figures given (*SD* in parenthesis)

In Poland, English acquisition occurs in a monolingual country; the NL is almost exclusively Polish, as only 1.55% of citizens identify with a national or ethnic identity other than Polish (Central Statistical Office of Poland, 2013). Polish students rarely come into contact with a language different from their NL outside school in everyday interactions, which is unique in Europe. EFL instruction begins early, either in the obligatory reception year (entered usually at 6 years) or in an optional kindergarten. Polish is used for all course instructions, while English is used only for EFL class, and often combined with Polish (Gajewska-Dyszkiewicz et al., 2011). NL literacy instruction is based on an analytic-synthetic method (Awramiuk & Krasowicz-Kupis, 2014), combined with a global one (Jaszczyszyn, 2010). The teaching strategy is based on sound and/or syllable segmentation and blending is an effective strategy for Polish (Awramiuk, 2006). In EFL instruction, students are expected to already be able to decode and/or recognise words. In accordance with the state-wide core curriculum, the number of EFL instruction hours per week in a 3-year cycle was exact number of hours not specified, approximately 6 h/week in elementary school year 1 to 3 (i.e. 2 h/week each year), 8 h/week in elementary school year 4 to 6, 9 h/week in junior high school, and 15 h/week (for 2 foreign languages combined) in high school (Minister of Sport and National Education, 2002). Ninety-four percent of junior high school and 98% of high school students study English (Braunek, 2013).

All students in the criterion group had been identified as demonstrating dyslexia. A legally valid report following the ICD-10 (World Health Organisation, 1992) had been issued independently by state and non-state psychological and educational counselling centres. Diagnostic criteria included IQ over 85, achievement test scores for reading and spelling below the − 1 SD cutoff (decoding, text reading, reading comprehension, writing), and processing deficit symptoms including phonological skills, assessed with standardised testing measures. Deficits need not be manifested in all tests. This prior assessment was confirmed (Table 1), as students with dyslexia, compared with their normally reading peers, read single Polish nonwords less accurately and more slowly, and exhibited deficits in phoneme blending and RAN. The groups did not differ in verbal and phonological short-term memory and phoneme segmentation. Moreover, students with dyslexia, as compared with their normally reading peers, read single English words and nonwords less accurately and more slowly (Table 1).

### Materials and methods

#### Questionnaire

A short survey developed by the authors was used to collect demographic data, as well as information about educational level, English instruction and exposure, and the diagnosis of dyslexia. A separate version was completed by the students and their parents.

#### NVR

The Raven’s Matrices (1991), a Polish adaptation, was used to match the groups for the intelligence level. Reliability was from rtt = 0.89, SEM = 2.37 to rtt = 0.94, SEM = 2.38.

### Reading measures in NL

Real word reading was measured using a task by Krasowicz-Kupis (Jaworowska, Matczak, & Stańczak, 2010). It assesses the accuracy (as measured with the number of words read correctly) and fluency (as measured with the time of reading) of decoding 89 unrelated Polish real words. Syllable blending errors and self-corrections were treated as errors, following the test manual. A Cronbach’s alpha for accuracy was .96.

Nonword reading was measured using a task by Bogdanowicz (Jaworowska et al., 2010). It assesses the accuracy (as measured by the number of errors) and fluency (as measured by the number of nonwords read within 1 min) of decoding 71 unrelated Polish nonwords. Syllable blending errors and self-corrections were not treated as errors, following the test manual. A Pearson’s *r* coefficient for test-retest reliability was .93.

### Reading measures in EFL

These tasks were developed by the authors for this study, to address a lack of tasks standardised for Polish population. The development process and more detailed characteristics of the tasks and their rationale were published in Łockiewicz & Jaskulska, 2016.

#### Real word reading task

This task assesses the accuracy (as measured by the number of words read correctly) and fluency (as measured by the number of words read within 1 min) of decoding 70 unrelated English real words. Self-corrections were not treated as errors, as the task was performed in EFL. A Cronbach’s alpha for accuracy was .88.

#### Nonword reading task

This task assesses the accuracy (as measured by the number of nonwords read correctly) and fluency (as measured by the time of reading) of decoding 30 unrelated English nonwords. Self-corrections were not treated as errors, as the task was performed in EFL. A Cronbach’s alpha for accuracy was .78.

### Phonological processing measures in NL

*Digit Span - Wechsler Memory Scale III* was measured using a Polish adaptation by Pąchalska and Lipowska (2006). In calculating the raw score, we used a composite total score (for forward and backward task) tapping verbal short-term memory. A Cronbach’s alpha was 0.83.

#### Linguistic skills

*Phonological memory task* was measured using a nonword repetition task (Bogdanowicz, Kalka, Karpińska, Sajewicz-Radtke, & Radtke, 2012). It assesses phonological short-term memory. Score is 1 point for every repeated Polish nonword, given in series consisting of 3 to 6 nonwords (Max = 18 points, sample item: *moleno*).

Phonological awareness in Polish was measured with a *phoneme segmentation and blending* (Bogdanowicz et al., 2012). This task used 16 Polish nonwords, which consisted of 5 to 12 phonemes. A raw score of 1 point was given for each nonword segmented into phonemes (Max = 8 points, sample item: *pakor*) for phoneme segmentation and 1 point for each nonword blended from phonemes (Max = 8 points, sample item: *w-a-r-y-n-o-l-e*) for phoneme blending. Full and segmented nonwords were given orally by an experimenter. A Cronbach’s alpha for accuracy was 0.659.

*Rapid automatised naming (RAN)* was measured using two tasks: (1) picture naming and (2) picture, letter, and digit naming (Bogdanowicz et al., 2012). In the former task, students were asked to name as fast as they could all items in a set of 42 colourful pictures that consisted of six pictures repeated on a page. In the latter task, the students were asked to name as fast as they could 42 pictures (identical to the six used in task 1), letters, and digits. In calculations, we used a composite total score (as measured with time in seconds) tapping RAN.

### Procedure

All participants completed two parts of the test, a 50-min group session (e.g. the questionnaire, the Raven Test Matrices), conducted by two researchers, and a 25-min individual session (e.g. words and nonword reading in NL and FL, verbal and phonological short-term memory, phoneme segmentation and blending, RAN measures), conducted by one researcher. All children completed the tasks in the same order. The instructions were given in Polish for all tasks to facilitate comprehension. The students were explicitly told when to answer in English; the tasks were grouped by language. The assessments were conducted at schools. All students and their parents expressed informed consent for the children to participate in the study.

## Results

The preliminary analyses regarding the assumption of normality of the distribution of statistics in the sample showed that the data allows for usage of parametric statistics (see Norman, 2010). There were no extreme outliers that could affect the analyses and the distributions did not show excessive skewness. To examine the associations between the variables, Pearson’s product-moment, point-biserial, and phi correlation coefficients were calculated (Table 2). Out of the five examined NL phonological processing skills, three—verbal short-term memory, phoneme blending, and RAN—correlated with NL and FL reading, while two—phonological short-term memory and phoneme segmentation—did not.

**Table 2:** Correlations between the study variables

Variable M(SD)/percentages		2.	3.	4.	5.	6.	7.	8.	9.	10.	11.	12.	13.	14.	15.	16.	17.
1. Dyslexia^ac^	45% dyslexic	− .074^d^	− .049	− .271**	− .473**	.453**	− .396**	.393**	.226**	.245**	.395**	− .210*	.139	.072	.008	.256**	− .459**
2. Educational level^bc^	30% junior		.475**	.157	− .433**	.103	.254**	.311**	.248**	.426**	.362**	− .129	.199*	.065	.047	.463**	− .184*
3. EFL instruction at school	8.43 (2.08)			.161	− .276**	.064	.193*	.173*	.284**	.408**	.223*	− .155	− .066	− .005	.060	.198*	− .210*
4. EFL private tutoring	2.38 (3.24)				.020	.073	.160	− .047	.163	.283**	.085	.083	.074	− .056	.128	.162	.195*
5. NLWF^e^	72.11 (20.57)					− .554**	.108	− .768**	− .585**	− .622**	− .576**	.538**	− .275**	− .126	− .122	− .511**	.568**
6. NLWA	82.74 (5.17)						− .433**	.495**	.329**	.540**	.569**	− .365**	.306**	.122	− .077	.377**	− .344**
7. NLNA^e^	8.48 (4.69)							− .260**	− .019	− .158	− .372**	.179*	− .267**	− .030	.043	− .060	.182*
8. NLNF	53.77 (11.80)								.474**	.575**	.668**	− .628**	.338**	.126	.091	.402**	− .451**
9. EFLWF	66.58 (7.56)									.777**	.452**	− .526**	.152	.065	.124	.324**	− .363**
10. EFLWA	55.94 (12.43)										.734**	− .410**	.302**	.143	.096	.486**	− .391**
11. EFLNA	20.17 (4.48)											− .416**	.343**	.161	.103	.509**	− .379**
12. EFLNF^e^	35.65 (10.38)												− .190*	− .150	− .106	− .249**	.352**
13. VM	13.16 (2.63)													.305*	.132	.307**	− .225**
14. PM	9.07 (2.86)														.186*	.110	− .126
15.PS	6.13(1.32)															.208*	− .017
16. PB	3.97 (1.74)																− .319**
17. RAN (in sec.)^e^	76.76 (20.97)																

**p ≤ .01; *p ≤ .05; Pearson product-moment correlations, except: ^c^point-biserial correlation coefficients, ^d^phi correlation coefficient

^a^1 = dyslexia, 2 = control, ^b^1 = junior high school, 2 = high school, ^e^higher score signifies worse performance. *EFLWF*, EFL (English as a Foreign Language) word reading fluency; *EFLWA*, EFL word reading accuracy; *EFLNA*, EFL nonword reading accuracy; *EFLNF*, EFL nonword reading fluency; *NLWF*, NL (Native Language: Polish) word reading fluency; *NLWA*, NL word reading accuracy; *NLNA*, NL nonword reading accuracy; *NLNF*, NL nonword reading fluency; *VM*, verbal short-term memory; *PM*, phonological short-term memory; *PS*, phoneme segmentation; *PB*, phoneme blending

### The contribution of NL phonological processing skills to EFL reading skills

To test the hypothesis that phonological processing skills in NL (verbal and phonological short-term memory, phoneme blending, phoneme segmentation, RAN) contribute to EFL reading skills (word and nonword reading accuracy and fluency), several hierarchical multiple regression analyses were conducted. A diagnosis of dyslexia, educational level, and the length of EFL instruction were entered as independent variables in step 1; phonological processing skills in NL were entered as independent variables in step 2. EFL reading skills were entered as dependent variables (Table 3).

**Table 3:** Results of hierarchical regression analyses in which dyslexia, educational level, years of studying English as a Foreign Language (EFL) at school and privately, and phonological processing abilities in NL (Native Language: Polish) were regressed upon literacy in EFL

		Word reading		Nonword reading	
Step	Predictor	Fluency	Accuracy	Fluency^c^	Accuracy
1	Dyslexia^a^	.332 (3.97)**	.387 (5.30)**	− .269 (3)**	.505 (6.58)**
	Educational level^b^	.178 (1.94)*	.299 (3.73)**	− .130 (1.32)	.352 (4.17)**
	EFL at school	.189 (2.05)*	.243 (3.03)**	− .116 (1.18)	.061 (.72)
	EFL private tutoring	.207 (2.46)*	.304 (4.14)**	.031 (.35)	.140 (1.82)
	Δ*R*^2^	.214**	.402**	.107**	.346**
2	Dyslexia^a^	.183 (1.96)*	.214 (2.73)**	− .098 (.99)	.334 (4.13)**
	Educational level^b^	.078 (.76)	.120 (1.39)	.010 (.10)	.145 (1.63)
	EFL at school	.145 (1.54)	.250 (3.17)**	− .095 (.95)	.086 (1.06)
	EFL private tutoring	.201 (2.32)*	.274 (3.78)**	.046 (.51)	.082 (1.10)
	Verbal memory	− .031 (.34)	.082 (1.07)	− .033 (.34)	.094 (1.20)
	PM	.004 (.05)	.067 (0.95)	− .094 (1.06)	.088 (1.21)
	PS	.091 (1.12)	− .014 (0.20)	− .055 (.63)	.005 (.07)
	PB	.139 (1.40)	.238 (2.87)**	− .174 (1.67)	.314 (3.68)**
	RAN (in sec.)^c^	− .239 (2.47)*	− .167 (2.05)*	.222 (2.18)*	− .097 (1.17)
	Δ*R*^2^	.072*	.098**	.097*	.123**
	Total *R*^2^/Adj. *R*^2^	.286/.232*	.499/.461**	.204/.143*	.469/.429**

**p ≤ .01, *p ≤ .05

*β* given (*t* in parenthesis); ^a^1 = dyslexia, 2 = control, ^b^1 = junior high school, 2 = high school; ^c^higher score signifies worse performance; *PM* phonological short-term memory, *PS* phoneme segmentation, *PB* phoneme blending

The regression analysis for word reading fluency in EFL showed that the independent variables explained a total of 29% of the variance (*F*_9,118_ = 5.26, *p* ≤ .001). Significant independent variables in step 2 were a diagnosis of dyslexia (*β* = .18), showing that participants without dyslexia scored higher, years of studying EFL privately (*β* = .20), and RAN (− .24) (see Table 3).

The regression analysis for word reading accuracy in EFL showed that the independent variables explained a total of 50% of the variance (*F*_9,118_ = 13.08, *p* ≤ .001). Significant independent variables in step 2 were a diagnosis of dyslexia (*β* = .21), showing that participants without dyslexia scored higher, years of studying EFL at school (*β* = .25) and privately (*β* = .27), phoneme blending (*β* = .24), and RAN (− .17) (see Table 3).

The regression analysis for nonword reading fluency in EFL showed that the independent variables explained a total of 20% of the variance (*F*_9,117_ = 3.33, *p* ≤ .001). A significant independent variable in step 2 was RAN (*β =* .22), showing that participants who named visual material faster scored higher (see Table 3).

The regression analysis for nonword reading accuracy in EFL showed that the independent variables explained a total of 47% of the variance (*F*_9,117_ = 11.05, *p* ≤ .001). Significant independent variables in step 2 were a diagnosis of dyslexia (*β* = .33), showing that participants without dyslexia scored higher, and phoneme blending (*β* = .31) (see Table 3).

### NL reading skills as mediators in the relationship between NL phonological processing and EFL reading skills in the dyslexia group

**Fig. 1:**
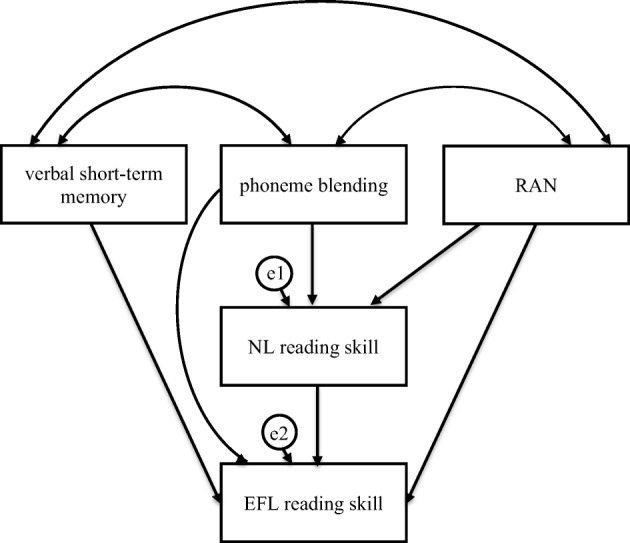
Mediation model in which verbal short-term memory, phoneme blending, and RAN were independent variables, NL (Native Language—Polish) reading skill was mediator, and EFL (English as Foreign Language) was dependent variable—initial model of relationships

**Fig. 2:**
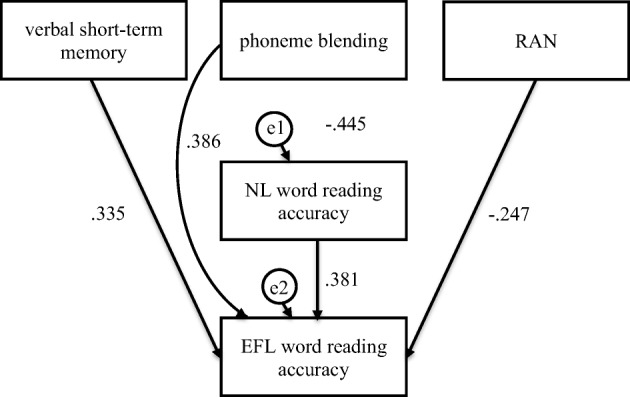
Mediation model in which verbal short-term memory, phoneme blending, and RAN were independent variables, Native Language (NL—Polish) word reading accuracy was mediator, and English as Foreign Language (EFL) word reading accuracy was dependent variable in the dyslexia group

**Fig. 3:**
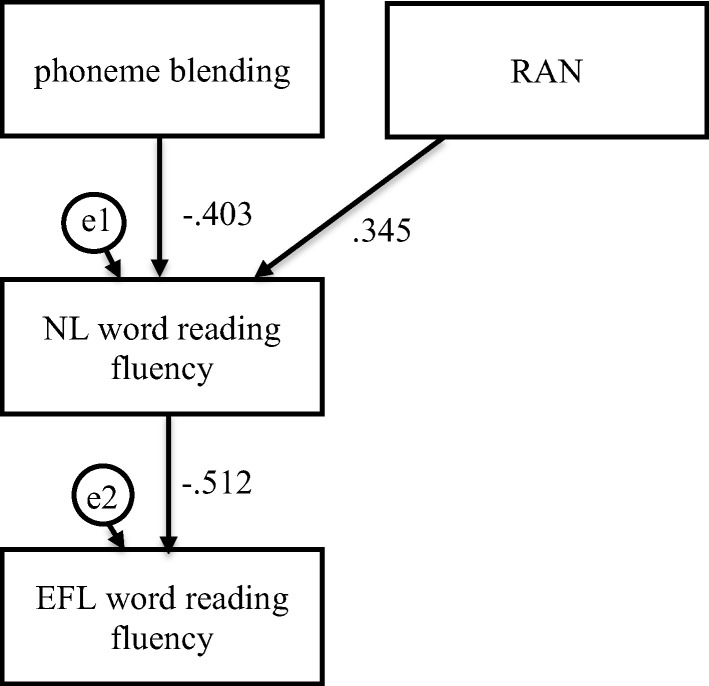
Mediation model in which phoneme blending and RAN were independent variables, Native Language (NL—Polish) word reading fluency was mediator, and English as Foreign Language (EFL) word reading fluency was dependent variable in the dyslexia group

**Fig. 4:**
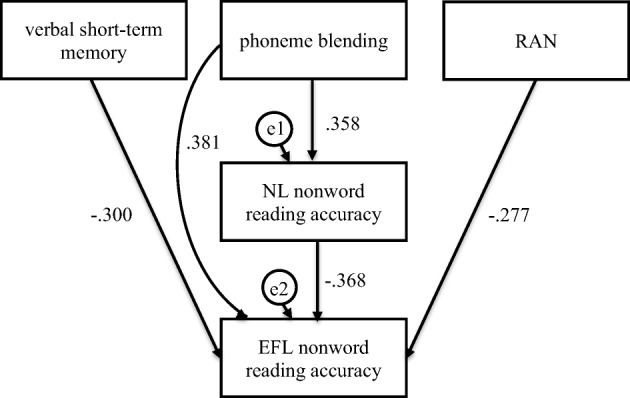
Mediation model in which verbal short-term memory, phoneme blending, and RAN were independent variables, Native Language (NL—Polish) nonword reading accuracy was mediator, and English as Foreign Language (EFL) nonword reading accuracy was dependent variable in the dyslexia group

**Fig. 5:**
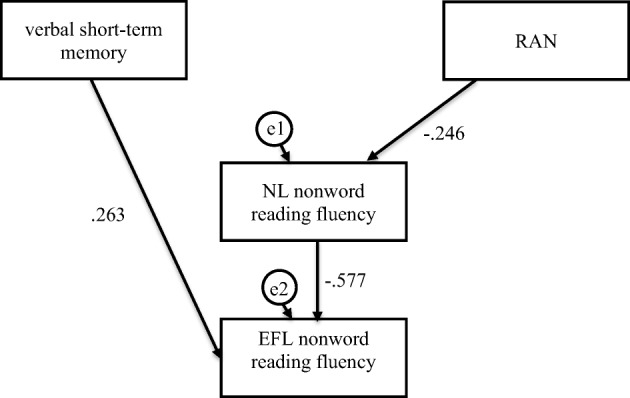
Mediation model in which verbal short-term memory and RAN were independent variables, Native Language (NL—Polish) nonword reading fluency was mediator, and English as Foreign Language (EFL) nonword reading fluency was dependent variable in the dyslexia group

**Fig. 6:**
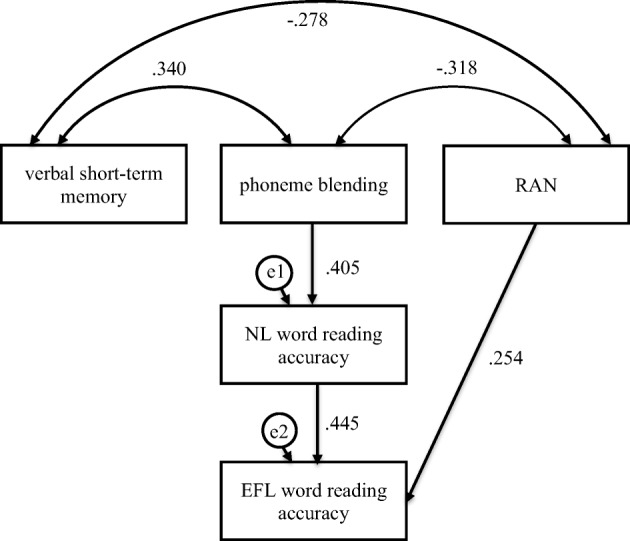
Mediation model in which verbal short-term memory, phoneme blending, and RAN were independent variables, Native Language (NL—Polish) word reading accuracy was mediator, and English as Foreign Language (EFL) word reading accuracy was dependent variable in the control group

**Fig. 7:**
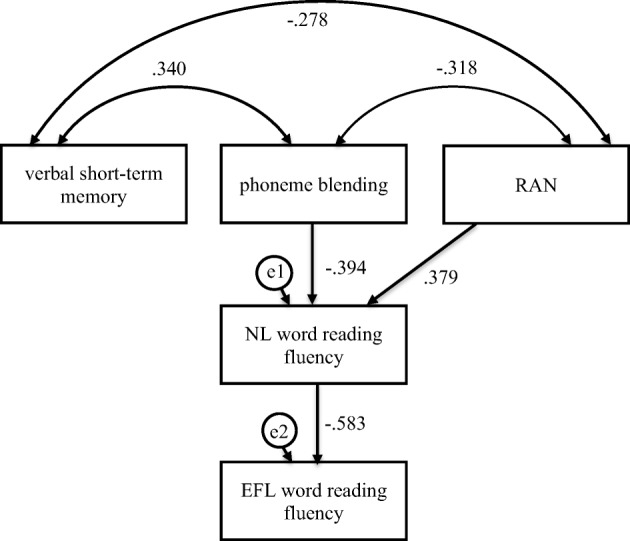
Mediation model in which verbal short-term memory, phoneme blending, and RAN were independent variables, Native Language (NL—Polish) word reading fluency was mediator, and English as Foreign Language (EFL) word reading fluency was dependent variable in the control group

**Fig. 8:**
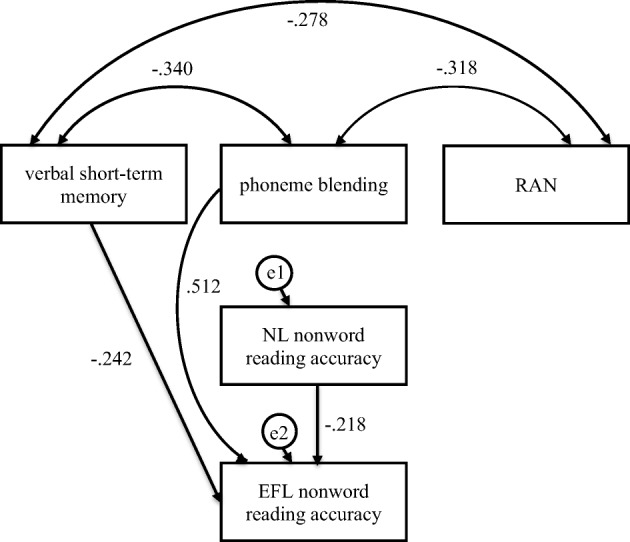
Mediation model in which verbal short-term memory, phoneme blending, and RAN were independent variables, Native Language (NL—Polish) nonword reading accuracy was mediator, and English as Foreign Language (EFL) nonword reading accuracy was dependent variable in the control group

**Fig. 9:**
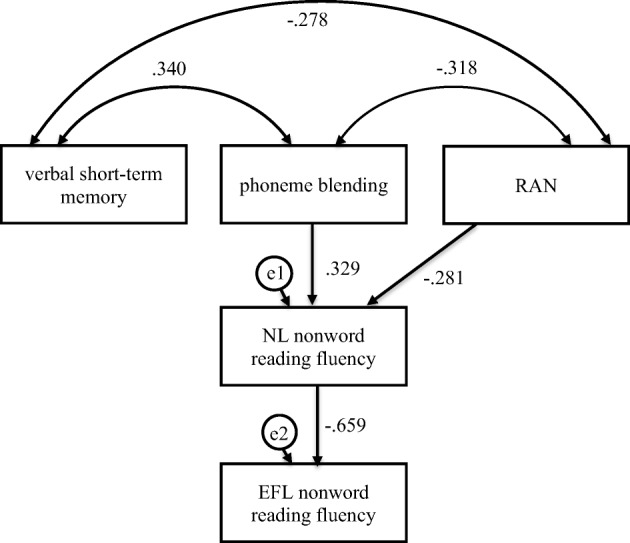
Mediation model in which verbal short-term memory, phoneme blending, and RAN were independent variables, Native Language (NL—Polish) nonword reading fluency was mediator, and English as Foreign Language (EFL) nonword reading fluency was dependent variable in the control group

To test the hypothesis that the relationship between the phonological processing skills in NL and word and nonword decoding and word recognition skills in EFL (word and nonword reading accuracy and fluency) is mediated by the accuracy and fluency of word and nonword decoding and word recognition skills in NL, we calculated mediation models using structural equation modelling. Bootstrap method with bias corrected 95% confidence intervals and 5.000 bootstrap samples were used. For each of the 4 dependent variables, we specified one mediation model. Based on the analysis of correlations (i.e. phonological short-term memory and phoneme segmentation did not correlate with NL and FL word and nonword decoding and word recognition skills), three of the examined NL phonological processing skills—verbal short-term memory, phoneme blending, and RAN—were entered as independent variables. NL and EFL reading skills entered in each mediation model were comparable, e.g. NL word reading accuracy as a mediator for EFL word reading accuracy. The analyses were calculated separately for the criterion and control group, as correlation and regression analyses showed that dyslexia is linked to EFL reading. Therefore, through using these separate analyses, we could have assessed more precisely the relationship between NL phonological processing and both word and nonword decoding and word recognition strategies in adolescent readers with dyslexia and without dyslexia. Model fit was evaluated using conventional criteria: CMIN/DF, CFI, RMSEA, and TLI (Table 4). The initial model for each mediation analysis is presented in Fig. 1. The final models for each mediation analysis, in which insignificant correlations, direct and indirect effects were removed to achieve best model fit, are presented in Tables 5 and 6 and Figs. 2, 3, 4, 5, 6, 7, 8, and 9; these figures present standardised regression coefficients.

**Table 4:** CMIN/DF, CFI, RMSEA, LO90, UI90, TLI coefficients in the compared groups

	Fig.	CMIN/DF	CFI	RMSEA	LO90	UI90	TLI
FL word reading accuracy—dyslexia group	2	0.860	1.000	0.000	0.000	0.154	1.040
FL word reading fluency—dyslexia group	3	1.325	0.944	0.074	0.000	0.185	0.919
FL nonword reading accuracy—dyslexia group	4	0.683	1.000	0.000	0.000	0.147	1.126
FL nonword reading fluency—dyslexia group	5	0.695	1.000	0.000	0.000	0.190	1.063
FL word reading accuracy—control group	6	1.703	0.957	0.096	0.000	0.215	0.891
FL word reading fluency—control group	7	0.947	1.000	0.000	0.000	0.168	1.006
FL nonword reading accuracy—control group	8	0.967	1.000	0.000	0.000	0.170	1.006
FL nonword reading fluency—control group	9	1.294	0.986	0.062	0.000	0.192	0.964

**Table 5:** Unstandardised regression coefficients, critical ratios, significance, and 95% confidence intervals for indirect effect in paths in mediation models, in which English as a Foreign Language (EFL) word reading accuracy and fluency were entered as dependent variables in the dyslexia group

			*B*	*C.R.*	*p*	Indirect effect
NLWA	←	VM	0.787	2.751	.006**	VM: lower CI .052, upper CI .215, *p* = .002
EFLWA	←	NLWA	0.945	3.673	.001**	
EFLWA	←	PB	3.077	3.718	.001**	
EFLWA	←	RAN^d^	− 0.149	2.380	.017*	
NLWF^d^	←	PB	− 5.512	3.687	.001**	PB: lower CI .115, upper CI .318, *p* ≤ .001
NLWF^d^	←	RAN^d^	0.358	3.152	.002**	RAN: lower CI − .304, upper CI − .066, *p* = .010
EFLWF	←	NLWF^d^	− 0.220	4.618	.001**	
NLNA^d^	←	PB	1.151	3.139	.002**	PB: lower CI − .258, upper CI − .046, *p* ≤ .001
NLNA^d^	←	VM	− 0.706	2.631	.009**	VM: lower CI .036, upper CI .223, *p* = .005
EFLNA	←	NLNA^d^	− 0.262	3.086	.002**	
EFLNA	←	PB	0.870	3.195	.001**	
EFLNA	←	RAN^d^	− 0.048	2.490	.013**	
NLNF	←	RAN^d^	− 0.124	2.044	.041*	VM: lower CI − .319, upper CI .022, *p* = .054
NLNF	←	VM	1.274	2.180	.029*	RAN: lower CI .004, upper CI .280, *p* = .087
EFLNF^d^	←	NLNF	− 0.674	5.477	.001**	

As path b was the same for EFL word reading accuracy (NLWF as independent variable, EFLWF as dependent variable), it was presented in the table only once

*VM*, verbal short-term memory; *PB*, phoneme blending; *NLWA*, NL (Native Language: Polish) word reading accuracy; *EFLWA*, EFL word reading accuracy; *NLWF*, NL word reading fluency; *EFLWF*, EFL word reading fluency; ^d^higher score signifies worse performance

**p ≤ .01; *p ≤ .05

**Table 6:** Unstandardised regression coefficients, critical ratios, significance, and 95% confidence intervals for indirect effect in paths in mediation models, in which English as a Foreign Language (EFL) word reading accuracy and fluency were entered as dependent variables in the control group

			*B*	*C.R.*	*p*	Indirect effect
NLWA	←	PB	0.964	3.882	.001**	PB: lower CI .102, upper CI .262, *p* ≤ .001
EFLWA	←	NLWA	1.123	4.437	.001**	
EFLWA	←	PB	1.526	2.531	.011**	
NLWF^d^	←	PB	− 3.179	4.208	.001**	PB: lower CI .149, upper CI .326, *p* ≤ .001
NLWF^d^	←	RAN	0.314	4.048	.001**	RAN: lower CI − .321, upper CI − .133, *p* ≤ .001
EFLWF	←	NLWF^d^	− 0.183	6.291	.001**	
NLNA^d^	←	VM	− 0.325	2.191	.028*	VM: lower CI .006, upper CI .123, *p* = .051
EFLNA	←	NLNA^d^	− 0.250	2.321	.020*	
EFLNA	←	PB	1.290	5.465	.001**	
NLNF	←	PB	2.063	3.156	.002**	PB: lower CI − .333, upper CI .097, *p* = .004
NLNF	←	RAN^d^	− 0.181	2.692	.007**	RAN: lower CI .096, upper CI .298, *p* ≤ .001
EFLNF^d^	←	NLNF	− 0.506	7.698	.001**	

As path b was the same for EFL word reading accuracy (NLWF as independent variable, EFLWF as dependent variable), it was presented in the table only once

*VM*, verbal short-term memory; *PB*, phoneme blending; *NLWA*, NL (Native Language: Polish) word reading accuracy; *EFLWA*, EFL word reading accuracy; *NLWF*, NL word reading fluency; *EFLWF*, EFL word reading fluency; ^d^higher score signifies worse performance

**p ≤ .01; *p ≤ .05

For the participants with dyslexia, verbal short-term memory was indirectly and phoneme blending and RAN directly linked to EFL word reading accuracy (Fig. 2, Table 5). The participants who scored higher in verbal short-term memory read NL single words more accurately (*β* = .335, *C.R.* = 2.751, *p* = .006), which was linked to a more accurate word reading in EFL (*β* = .381, *C.R.* = 3.673, *p* ≤ .001). Confidence intervals for an indirect effect were above zero (from .052 to .215, *p =* .002). Phoneme blending (*β* = .386, *C.R.* = 3.718, *p* ≤ .001) and RAN (*β* = − .247, *C.R.* = 2.380, *p* = .017) were directly linked to more accurate EFL word reading. Verbal short-term memory, phoneme blending, RAN, and NL word reading accuracy explained 36% of variance in EFL word reading accuracy. Phoneme blending and RAN were indirectly linked to EFL word reading fluency (Fig. 3, Table 5). The participants who scored higher in phoneme blending (*β* = − .403, *C.R.* = 3.687, *p* ≤ .001) and RAN (*β* = .345, *C.R.* = 3.152, *p* = .002) read NL single words faster, which was linked to faster (*β* = − .512, *C.R.* = 4.618, *p* ≤ .001) word reading in EFL. Confidence intervals for an indirect effect were above (from .115 to .318, *p ≤* .001) and below zero (from − .304 to − .066, *p =* .010), respectively. No direct effects were observed. Phoneme blending, RAN, and NL word reading fluency explained 26% of variance in EFL word reading fluency.

For the participants with dyslexia, verbal short-term memory was indirectly and phoneme blending partially indirectly linked to EFL nonword reading accuracy (Fig. 4, Table 5). The participants who scored higher in verbal short-term memory (*β* = − .300, *C.R.* = 2.631, *p* = .009) and lower in phoneme blending (*β* = .358, *C.R.* = 3.139, *p* = .002) read NL single nonwords more accurately, which was linked to a more accurate (*β* = − .368, *C.R.* = 3.086, *p* ≤ = .002) nonword reading in EFL. Confidence intervals for an indirect effect were above (from .036 to .223, *p* = .005) and below zero (from − .258 to − .046, *p ≤* .001), respectively. However, direct effects also occurred, which suggested that phoneme blending (*β* = .381, *C.R.* = 3.195, *p* ≤ .001) and RAN (*β* = − .277, *C.R.* = 2.490, *p* = .013) were related to more accurate EFL nonword reading. Verbal short-term memory, phoneme blending, RAN, and NL nonword reading accuracy explained 26% of variance in EFL nonword reading accuracy. Verbal short-term memory and RAN were indirectly linked to EFL nonword reading fluency (Fig. 5, Table 5). The participants who scored higher in verbal short-term memory (*β* = .263, *C.R.* = 2.180, *p* = .029) and RAN (*β* = − .246, *C.R.* = 2.044, *p* = .041) read NL single nonwords faster, which was linked to faster (*β* = − .577, *C.R.* = 5.477, *p* ≤ .001) nonword reading in EFL. Confidence intervals for an indirect effect were below (from − .319 to − .022, *p* = .054) and above zero (from .004 to .280, *p* = .087). No direct effects were observed. Verbal short-term memory, RAN, and NL nonword reading fluency explained 33% of variance in EFL nonword reading fluency.

### NL reading skills as mediators in the relationship between NL phonological processing and EFL reading skills in the control group

For the participants without dyslexia, phoneme blending was partially indirectly linked to EFL word reading accuracy (Fig. 6, Table 6). The participants who scored higher in phoneme blending (*β* = .405, *C.R.* = 3.882, *p* ≤ .001) read NL single words more accurately, which was linked to a more accurate (*β* = .445, *C.R.* = 4.437, *p* ≤ .001) word reading in EFL. Confidence intervals for an indirect effect were above zero (from .102 to .262, *p ≤* .001). A direct effect (*β* = .254, *C.R.* = 2.531, *p* = .011) suggested that better phoneme blending was related to more accurate EFL nonword reading. Phoneme blending and NL word reading accuracy explained 35% of variance in EFL word reading accuracy.

Phoneme blending and RAN were indirectly linked to EFL word reading fluency (Fig. 7, Table 6). The participants who scored higher in phoneme blending (*β* = − .394, *C.R.* = 4.208, *p* ≤ .001) and RAN (*β* = .379, *C.R.* = 4.048, *p* ≤ .001) read NL single words faster, which was linked to faster (*β* = − .583, *C.R.* = 6.291, *p* ≤ .001) word reading in EFL. Confidence intervals for an indirect effect were above (from .149 to .326, *p ≤* .001) and below zero (from − .321 to − .133, *p ≤* .001), respectively. No direct effects were observed. Phoneme blending, RAN, and NL word reading fluency explained 34% of variance in EFL word reading fluency.

In the participants without dyslexia, verbal short-term memory was indirectly linked to EFL nonword reading accuracy (Fig. 8, Table 6). The participants who scored higher in verbal short-term memory (*β* = − .242, *C.R.* = 2.191, *p* = .028) read NL single nonwords more accurately, which was linked to a more accurate (*β* = − .218, *C.R.* = 2.321, *p* = .020) nonword reading in EFL. Confidence intervals for an indirect effect were above zero (from 0.006 to .123). No direct effect for verbal short-term memory was observed. Phoneme blending was directly linked to EFL nonword reading accuracy. The participants who scored higher in phoneme blending (*β* = .512, *C.R.* = 5.465, *p* ≤ .001) read EFL single nonwords more accurately. Verbal short-term memory, phoneme blending, and NL nonword reading accuracy word reading fluency explained 33% of variance in EFL nonword reading accuracy.

Phoneme blending and RAN were indirectly linked to EFL nonword reading fluency (Fig. 9, Table 6). The participants who scored higher in phoneme blending (*β* = .329, *C.R.* = 3.156, *p* = .002) and RAN (*β* = − .281, *C.R.* = 2.692, *p* = .007) read NL single nonwords faster, which was linked to faster (*β* = − .659, *C.R.* = 7.698, *p* ≤ .001) nonword reading in EFL. Confidence intervals for an indirect effect were below (from − .333 to − .097, *p =* .004) and above zero (from .096 to .298, *p ≤* .001), respectively. No direct effects were observed. Phoneme blending, RAN, and NL nonword reading fluency explained 43% of variance in EFL nonword reading fluency.

## Discussion

We found that dyslexia, years of studying EFL both at school and privately, NL phoneme blending and RAN predicted word reading accuracy in EFL. Moreover, dyslexia, years of studying EFL privately, and NL RAN predicted EFL word reading fluency. These findings confirm that difficulties in FL acquisition result from NL deficits (Ferrari & Palladino, 2007), for example phonological processing deficits (Palladino & Ferrari, 2008), as phoneme blending and RAN contributed to EFL reading even when dyslexia was controlled for. However, dyslexia remained a significant factor in step 2 of the analysis, which shows that it interferes with FL acquisition, which has already been reported for several languages (Lindgren & Laine, 2011; Palladino et al., 2013; van Sette et al., 2017). Moreover, our results provide further evidence that phonological awareness in NL predicts accuracy of reading in FL or L2 (Gál & Orbán, 2013). The predictive function of RAN for both the accuracy and fluency of word reading is consistent with earlier studies on NL reading in a variety of languages (Norton & Wolf, 2012), and with EFL, Dutch as NL study (Morfidi et al., 2007). However, in this latter study, unlike in ours, phoneme awareness did not influence the accuracy of word reading. We attribute the relationship between phoneme blending and word reading accuracy to our participants’ incomplete familiarity with the read words, despite having studied EFL for 8 years on average. Half of junior high school students do not achieve the expected A2 (waystage or elementary) proficiency level, while teachers frequently use Polish during a typical English lesson (Gajewska-Dyszkiewicz et al., 2011), which further diminishes students’ exposure to EFL. The more frequently certain words appear in speech and writing, the faster and more accurately students recognise them (Wang & Koda, 2007). Likely, when encountering an unfamiliar word, our participants employed a phoneme blending strategy that they had practised during NL reading instruction (Dobkowska, 2014).

We also found that dyslexia and NL phoneme blending predicted the accuracy, while NL RAN predicted the fluency of EFL nonword reading. Reliance on grapheme/phoneme correspondence is a typical strategy when reading unfamiliar lexical items (Coltheart, 2006; Ehri, 1994). Our results are also in accordance with findings that NL nonword reading fluency depends to a greater extent on RAN than on phonemic awareness and phonological memory (González-Valenzuela, Díaz-Giráldez, & López-Montiel, 2016), which we demonstrated also for EFL decoding in more advanced readers. Even though in our study dyslexia did not predict EFL nonword reading fluency, NL RAN, which is typically poor in learners with dyslexia did (Wolf et al., 2000). Moreover, in our study, the participants with dyslexia read EFL nonwords slower as compared with their peers without dyslexia. These findings demonstrated that students with dyslexia were at risk of having problems with fluent FL reading. Generally, our results showed relationships between phonological processing and FL word and nonword decoding and word recognition that are analogous to the ones observed for NL.

In our study, actual exposure to English and years of instruction predicted only real word reading, but not nonword reading. A report on teaching practices in Polish elementary schools reveals that the most practiced skill is vocabulary, as 95% of year IV to VI teachers declared that their students do vocabulary exercises during every class. However, only 63.11% of teachers reported the same for reading aloud, which diminishes students’ practice of reading skills and pronunciation (Muszyński, Campfield, & Szpotowicz, 2015). Our result suggests that English instruction in Poland is not effective enough in teaching diverse phoneme-grapheme correspondence rules in FL, crucial for nonword decoding. Likely, the EFL teachers concentrate on the meaning and spelling of words, not on their correct pronunciation. Additional phonetics exercises, which are largely overlooked, would help the students to realise and practise unfamiliar FL phoneme/grapheme correspondences.

In our study, verbal and phonological short-term memory did not predict EFL reading. As mental lexicon develops, learning new words in FL depends to a greater extent on long-term rather than short-term phonological memory (Masoura & Gathercole, 2005). As our learners had been studying English for 8 years on average, they likely used to a greater extent their mental lexicons and RAN skills, which rely, among other factors, on working memory (Norton & Wolf, 2012). Overall, our data support the theories about a direct access to known FL words while reading in this sample (Coltheart, 2006; Ehri, 1994).

We also found that certain NL phonological processing skills: verbal short-term memory, phoneme blending, and RAN contributed to EFL reading skills only through or partially through NL reading in both participants with and without dyslexia. In general, better NL phonological processing skills related to more accurate and fluent NL reading that, subsequently, facilitated EFL reading skills. We assume this order of influence as our participants had started NL literacy instruction prior to EFL literacy instruction. Moreover, their exposure to NL, both privately and academically, was substantially greater. Our results provide further evidence for the linguistic transfer of phonological skills (Geva & Verhoeven, 2000; Melby-Lervåg & Lervåg, 2011), and LCDH (Sparks et al., 2009; Sparks et al., 2006).

Generally, in our study, the patterns of relationships between NL phonological processing, NL serving as a mediator, and EFL reading skills were most similar in the dyslexia and the control group for tasks measuring reading fluency, which tap, among other abilities, the speed of processing (Kail & Hall, 1994). Specifically, we found that in both groups NL word reading fluency mediated the relationship between phoneme blending, RAN, and EFL word reading fluency. Similarly, Morfidi et al. (2007) found that speeded word reading in Dutch (NL) predicted speeded word reading in EFL. Moreover, in the control group, we observed identical relations for the NL nonword reading fluency, which mediated the relationship between phoneme blending, RAN, and EFL nonword reading fluency. Well-developed speeded NL reading, which depends on phoneme blending (Mather & Wendling, 2012) and RAN (Norton & Wolf, 2012; Wolf et al., 2000) skills, relates to the pace of FL word reading in both students who are normal and slow readers. In fact, RAN strongly correlates with the speed of decoding (Poulsen, Juul, & Elbro, 2015), a relation that we confirmed also for EFL reading. In the dyslexia group, though, NL nonword reading fluency mediated the relationship between RAN and verbal short-term memory (the latter link was not observed for the control group) but not phoneme blending, and EFL nonword reading fluency. However, both RAN and reading rely on working memory (Norton & Wolf, 2012). Moreover, we found that in both groups, all NL phonological processing skills related to EFL reading fluency only when NL reading fluency was entered as a mediator, which suggests that practice in NL reading fluency facilitates EFL reading fluency.

In our study, the patterns of relationships between NL phonological processing, NL serving as a mediator, and EFL reading skills were more different in the dyslexia and the control group for tasks measuring word and nonword reading accuracy, which tap, among other abilities, letter-to-sound conversion (Melby-Lervag, Lyster, & Hulme, 2012), than for tasks measuring word and nonword reading fluency. We found that in the control group, NL word reading accuracy mediated between phoneme blending (partially) and EFL word reading accuracy. In the dyslexia group, however, NL word reading accuracy mediated the relationship between verbal short-term memory and EFL nonword reading fluency, but phoneme blending and RAN were only directly related to EFL word reading accuracy. Therefore, only the participants without dyslexia employed their phoneme blending skills both directly and through their more developed NL when faced with an EFL reading task. The participants with dyslexia relied on their verbal short-term memory, which is characteristic for an earlier stages of FL acquisition (Masoura & Gathercole, 2005).

Similarly, in our study, NL nonword reading accuracy mediated the relationship between verbal short-term memory and EFL nonword reading accuracy in both the participants with and without dyslexia. This was arguably the most difficult task, as the students had to employ only the English letter-to-sound correspondence rules, which are very different from Polish ones (Jaskulska & Łockiewicz, 2017; Nijakowska, 2010). Thus, the participants had to override the dominant response of applying NL letter-to-sound matching, shift between NL and FL rules, and update and monitor working memory contents, which reflects 3 aspects of executive functions in Miyake and Friedman’s model (2012). Likely, this is why our participants relied on verbal short-term memory, measured with two tasks, one of which also tapped working memory (i.e. repeating a series of digits backwards, which requires the dual task of simultaneous storage and manipulation of the given material) (cf. Alloway, Gathercole, & Pickering, 2006). The phonological memory task, which did not correlate with reading measures, did not tap working memory, as it relied on simple repetition of nonwords in an unchanged form, relying only on storage, but not on manipulation of the given material (cf. Alloway et al., 2006).

Moreover, our results indicated that in the dyslexia group, NL nonword reading accuracy mediated the relationship between phoneme blending (partially). In addition, RAN related to EFL nonword reading accuracy directly in the dyslexia group, and phoneme blending related to EFL nonword reading accuracy directly in the control group. Similarly, nonword reading in English (NL) predicted proficiency in Spanish (FL) (Meschyan & Hernandez, 2002). However, paradoxically, in the dyslexia group, the participants who scored higher in phoneme blending read NL nonwords less accurately, but EFL nonwords more accurately, as expected. In addition, better NL phoneme blending was directly related to more accurate EFL nonword reading. The explanation for this finding is not clear; however, Miller-Guron and Lundberg (2000) suggested a difference in nonword decoding strategies between Swedish students with dyslexia who preferred to read in Swedish and those who preferred to read in English, which could support a Dyslexic Preference for English Reading phenomenon. As we did not ask our participants about their reading preferences, we cannot investigate if our dyslexic group included students who preferred to read in English. We would like to examine this issue in later studies.

In future studies, we intend to examine the relationship between NL phonological processing skills, and other NL and EFL literacy skills, including grammar, listening, and reading comprehension, and to conduct further large scale studies with a higher number of participants which would allow for complex multivariate analysis. We would also like to compare errors committed in reading by English (native speakers) and Polish students with dyslexia, to investigate possible differences in the patterns of difficulties. In the present study, quite unexpectedly, phoneme segmentation did not relate with reading measures; only phoneme blending did. Though segmentation is more related to spelling, and blending to reading (Mather & Wendling, 2012), which could explain the results in our study, this issue requires further analysis in future studies, with a wider array of segmentation and blending measures.

In our study, for 3 out of 4 of the compared skills: EFL nonword reading accuracy, EFL word and nonword reading fluency, NL phonological processing skills, and a counterpart NL reading skill consistently explained more variance in EFL performance in the control group, as compared with the dyslexia group (difference of 7%, 8%, and 11%, respectively). This result suggests that when faced with familiar and unfamiliar lexical material that does not follow phonotactic constraints of NL, learners with dyslexia benefit less from their NL skills when learning FL, as compared with their peers without dyslexia. Thus, proficiency in the accuracy of NL word decoding and recognition and in the fluency of NL nonword decoding contribute more to FL reading in normally developing than in slow readers, even when the two languages differ in orthographic consistency, as the grapheme-phoneme correspondence rules are much more inconsistent for English than for Polish (Awramiuk, 2006; cf. Ziegler & Goswami, 2005). Positive linguistic transfer (cf. Odlin, 1989) seems then to be less influential in individuals with dyslexia, as compared with individuals without dyslexia, in FL acquisition.

Summing up, our results demonstrate that NL word and nonword decoding and word recognition skills mediated the relationship between NL phonological processing and EFL word and nonword decoding and word recognition skills, though this relationship was more conspicuous in the control group, as compared with the dyslexia group. The pattern of relations between NL phonological processing, NL reading, and EFL reading was similar for reading fluency, but not for reading accuracy in the compared groups. This relation was observed for a NL (Polish) and FL (English), which differ in the consistency of grapheme-phoneme correspondences. In addition, 2 out of 3 examined NL phonological processing skills: phoneme blending and RAN (with the exception of verbal short-term memory) that predicted EFL reading skills were more poorly developed in the participants with dyslexia, as compared with their peers without dyslexia. This finding underlines the difficulties that students with dyslexia face when learning FL.

## Conclusions

Our results show that despite the substantial differences in grapheme-phoneme correspondence patterns and the resulting dissimilar methods of reading instruction, NL word and nonword decoding and word recognition skills mediate the relationship between NL phonological processing skills and EFL word and nonword decoding and word recognition skills. However, the pattern of relations is similar for reading fluency, but not for reading accuracy in the participants with and without dyslexia. Moreover, this relationship is more conspicuous in the control group, as compared with the dyslexic group, which suggests that normal readers benefit more from their NL reading skills when learning to read in FL. As in the majority of European countries first FL instruction begins between 5 and 9 age range (Enever, 2012), NL proficiency should be used as an additional tool to facilitate FL learning. In Poland, remedial teaching classes are centred on developing NL literacy skills, which seem to contribute less to the development of FL literacy skills of students with dyslexia. Thus, we believe that when students with dyslexia struggle with FL literacy, they should attend remedial teaching classes that would be focused specifically on FL.
